# Zoonotic Parasites and Their Association With Human Activities in Northern Tanzania: An Integrated Ecosystem Approach for One Health

**DOI:** 10.1155/2024/8872837

**Published:** 2024-07-19

**Authors:** Barakaeli Abdieli Ndossi, Eblate Ernest Mjingo, Hansol Park, Dongmin Lee, Mohammed Mebarek Bia, Heejae Yang, Sungbo Seo, Keeseon S. Eom

**Affiliations:** ^1^ Tanzania Wildlife Research Institute, P.O. Box 661, Arusha, Tanzania; ^2^ Department of Parasitology Parasitology Research Center and International Parasite Resource Bank Chungbuk National University School of Medicine, Cheongju 28644, Republic of Korea; ^3^ Division of Biobanking Solutions Cocoon Inc. 116 194-41, Uiryodanji-gil, Osong-eup, Heungdeok-gu, Cheongju-si, Chungcheongbuk-do, Republic of Korea

**Keywords:** awareness, Northern Tanzania, public health, soil-transmitted helminths, zoonosis

## Abstract

The community's awareness of the prevalence and impact of zoonotic diseases has been significantly underestimated, leading to insufficient implementation of control measures. This study was carried out in Northern Tanzania between 2019 and 2023 to investigate zoonotic parasites and the risks associated with human activities that contribute to zoonotic diseases. Cross-sectional surveys were conducted in 12 villages, including nine in Loliondo Division and three in Babati District. Focus Group Discussions and Key Informant Interviews were conducted to assess the community's knowledge and practices regarding the risks associated with zoonotic diseases in the surveyed areas. A total of 255 samples were collected from various sources, including latrines, households, livestock enclosures, domestic dogs, and chickens. Out of these samples, 152 tested positive for identifiable parasite eggs and oocysts. These parasites included hookworms (21.7%), *Trichuris* sp. (14.5%), *Strongyloides* sp. (13.8%), *Eimeria* sp. (19.7%), Taeniids (5.9%), *Hymenolepis* sp. (3.3%), *Spirometra* sp. (2.6%), and *Dipylidium* sp. (0.7%). Taeniids and *Spirometra* species were predominantly found in villages near protected areas such as Arash Sokoni, Oloipiri, Sukenya, Wasso, Orkuyiene, Haytemba, and Loliondo. Hookworms were most commonly detected in Arash Sokoni, Loliondo, Isuguro, and Hyatemba, while *Strongyloides* sp. was prevalent in Wasso, Sukenya, and Olobo villages. The quantitative analysis reveals significant associations between hygiene practices, proximity to livestock enclosures, ecological factors, and human–animal interaction, highlighting their pivotal roles in determining soil-transmitted helminth (STH) prevalence across different villages. This study reveals that there was a generally low level of awareness regarding zoonotic diseases and STHs. The detection of STH samples, combined with the limited understanding of zoonotic diseases, emphasizes the importance of taking proactive measures to reduce transmission risks. Prioritizing education and promoting awareness along with implementing comprehensive strategies are essential steps to effectively tackle the problems linked to STH infections and substantially lessen the public health burden caused by zoonotic diseases.

## 1. Introduction

The prevalence of newly emerging pathogens and zoonotic diseases has increased over recent decades due to factors such as climate change, habitat manipulation, human lifestyle, and spatial distribution of animals [1–3]. Zoonotic diseases encompass a broad spectrum of pathogens that have significant global health impacts with an estimated 2.5 billion cases and 2.7 million deaths in low- and middle-income countries [4–7]. Soil-transmitted helminths (STHs) such as *Ascaris lumbricoides*, *Ancylostoma duodenale*, *Necator americanus*, *Trichuris trichiura*, and *Strongyloides stercoralis* pose significant health risks to human leading to conditions like anemia, impaired cognitive development, stunted growth, and mortality [8, 9].

Prior epidemiological evidence suggests the zoonotic potential of STHs, indicating a complex interaction between humans and animals in the transmission dynamics [10–12]. Additionally, recent studies have identified zoonotic transmission routes, particularly in regions with close human–animal interactions [13–15]. These findings highlight the importance of understanding the broader ecological context in which zoonotic diseases, including STH, operate, emphasizing the need for comprehensive studies surrounding human, animal, and environmental factors.

However, the community's perception on the prevalence and impact of zoonotic diseases has been critically underestimated, leading to inadequate implementation of control strategies [16]. In Northern Tanzania, there is a limited level of knowledge and awareness among communities regarding the risk factors for zoonotic diseases including transmission routes and preventive measures.

The rationale for an increase of zoonotic diseases is attributed to the complex life cycle of zoonotic parasites, involving multiple host species and the dynamics of human–wildlife interactions [17, 18]. Additionally, poor sanitation practices, lack of knowledge, and adherence to traditional beliefs contribute to the prevalence of zoonotic diseases [19, 20]. However, there has been limited investigation into epidemiology of various zoonotic diseases among pastoral and agropastoral communities in Northern Tanzania. As a result, the medical, public health, and veterinary sectors operate independently, resulting to fragmented and uncoordinated efforts to control zoonotic diseases [21, 22].

Therefore, it is crucial to undertake a comprehensive study on zoonotic diseases in the northern region of Tanzania to establish fundamental data aimed at enhancing public health outcomes and mitigating risks associated with zoonotic diseases.

This study is aimed at establishing a fundamental understanding of the prevalence of zoonotic parasites and the health risk factors associated to human activities in Northern Tanzania.

The primary objective is to assess the level of understanding the health risk factors among individuals and obtain baseline information associated with activities conducted in close proximity to protected areas. The outcomes of this study will contribute to the development of effective interventions and strategies for the prevention and control of zoonotic diseases in Tanzania. This will enhance holistic improvement to the community welfare by strengthening understanding of zoonotic disease transmission dynamics and advocating for behavioral changes conducive to the adoption of preventive measures.

## 2. Materials and Methods

### 2.1. Study Area

This study was conducted in the selected villages constituted in Loliondo Division and Babati District in Northern Tanzania. Twelve (12) villages were surveyed, with nine in Loliondo Division and three in Babati District (Table 1).

The choice of villages in this study relied on human activities involving interactions with wildlife and livestock, considering farmers and ethnic composition. The predominant ethnic group consisted of Maasai (90%) in Loliondo Division and Iraqw resided in Babati District.

### 2.2. Data Collection

#### 2.2.1. Focus Group Discussions

Focus Group Discussions were conducted in all study areas (*n* = 12) to explore views and common practices linked to zoonosis and STH. The survey was focused on the knowledge on zoonosis, causes, symptoms, transmission, and history of zoonosis. The community perception on the STH, accessibility, and availability of health services related to STH and zoonotic disease were also assessed. In addition, the success, challenges, failure, and sustainability of any medical services were asked.

Each survey and conversations were accompanied by a research assistant using the local language that is understandable by all participants to ensure that they are comfortable to share their views and suggestions. A structured interview was deployed accompanied with the guiding questions while taking notes for all discussions.

#### 2.2.2. Key Informant Interviews (KII)

A total of six KIIs from each village were considered based on the community selection. Village elder, veterinary officer, human health officer, field livestock officer, pastoralist leader, and women elder in the community with profound information were purposely selected for participation. Using a semistructured interview guide, the information collected was general knowledge on zoonosis and STH based on the causes and transmission, health-seeking behavior of community, and general information about the human interaction with the wildlife species.

#### 2.2.3. Knowledge, Attitudes, and Practice Surveys

For the monitoring survey, a questionnaire was used to collect information about the knowledge, attitudes, and practices of community members on the perception of the human interaction toward wildlife species and the risk of zoonotic diseases and STH. In addition, sociodemographic information of study participants was collected.

#### 2.2.4. Parasitological Survey for STHs

A total of 255 samples were collected from latrines, households, livestock enclosures, domestic dogs and chickens that were collected in 12 villages. For the purpose of this study, a livestock enclosure refers to livestock cages where pastoralists keep their livestock after grazing. Fecal samples (10 g) were collected in duplicate sterilized containers preserved under 10% formalin and 70% ethanol for further procedures. Soil sampling was considered in three locations such as livestock enclosures, latrines, and households from February 2019 to 2023. Hundred grams (100 g) of soil were collected in the depth of 3–10 cm from the surface by using a sterilized soil sample probe of 12 in., placed in a polythene zip bag, duplicated, labelled, and brought to the laboratory at the Wasso Hospital, Loliondo, and the Parasitology Laboratory in Tanzania Wildlife Research Institute (TAWIRI) resided in Serengeti National Park.

### 2.3. Diagnostic Method

#### 2.3.1. Floatation Method

Floatation method was deployed following Zenner et al. [23] and Steinbaum et al. [24] with some modifications. Briefly, 10 g of soil samples was harmonized and mixed in 50 mL of surfactant (five drops of liquid detergent per 1 L of water). Similar procedures were performed in fecal samples. The mixture was left for 30 min to settle and then poured through a double layer of gauze to remove the large particles. The resulting mixture was then transferred to a test tube and centrifuged at 1500 rpm for 10 min. The supernatant was gently poured off without disturbing the soil pellet and mixed with Sheather's sugar solution (550 g of table sugar in 450 mL of sterilized water and stirred until dissolved). The mixture was shaken vigorously and centrifuged at 1500 rpm for 10 min. The supernatant was then transferred to a 10 mL tube. For qualitative analysis, the tube was filled with floatation solution up to the upper meniscus, and a coverslip was carefully placed on the meniscus to collect the topmost portion. Around 20 min later, the coverslip was removed, placed onto a glass slide, and examined for the presence of parasitic eggs using a light microscope. Five milliliters of 10% formalin was added to the prepared solution to avoid mould contamination solution [25].

#### 2.3.2. Formal-Ether Sedimentation Method

Formalin ether sedimentation method was used according to Uga et al. [26] and Sato et al. [27] with some modifications. Prior to observation, 10 g of the collected soil samples was homogenized in the collection tube. Similar procedures were performed in fecal samples. After homogenization, they were separated into 1 g and prepared for sedimentation. The pellet mixtures were mixed with 7 mL 10% formalin in a 15 mL conical bottom polypropylene tube (Falcon®-type tube). Three milliliters of ether was added, and the solution was shaken vigorously for at least 60 s releasing the air pressure in the tube occasionally. The mixture was centrifuged for 10 min at 15000 rpm. The ring of floating detritus and then the formalin were decanted. The sedimentation process was repeated twice to clarify the sediment. The remaining pellet was suspended in formol-saline up to 200 *μ*L. From this suspension, 30 *μ*L was examined on a slide under a light microscope in a complete and systematic way. For each sediment sample, two slides (coverslip of 24 × 32 mm) were examined under light microscopy on the same day.

### 2.4. Data Analysis

Data obtained from interviews were used to assess the correlation between various aspects of community lifestyle (behavioral factors, ecological, human–animal interaction, population density of wild and domesticated animals, species diversity, and knowledge about STH or zoonotic potential) and zoonotic risks in the surveyed villages. Statistical analyses, including chi-square tests, were employed to determine significant associations, with a *p* value threshold of < 0.05, using Microsoft Excel Office version 2016.

## 3. Results

The demographic information of the surveyed participants is summarised in Table 2. A total of 35 individuals (males = 22, females = 13) participated in the survey. Out of the overall participant count, 24 were residents of Loliondo Division and 11 were from Babati District. The selection of respondents was based on specific criteria, including prevalence, historical context, and proximity to protected areas. In each of the five chosen wards, seven respondents were selected, taking into account their age, occupation, and educational level. Gender distribution was also considered during the survey, resulting in a male-to-female ratio of 2 : 1. The age range of participants was diverse, encompassing both those under 30 years old and those above 30 years old throughout the entire survey period (see Table 2). The majority of male participants in Loliondo Division (81.16%) were engaged in pastoralist activities, primarily raising cattle, sheep, goats, and dogs. In contrast, Babati District had a higher presence of sheep, goats, chickens, and dogs. Regarding education, it was observed that a significant portion of participants had completed primary-level education, while 25% of them had received secondary or tertiary education.

### 3.1. Coprological Examination of Parasites

**Figure 1:**
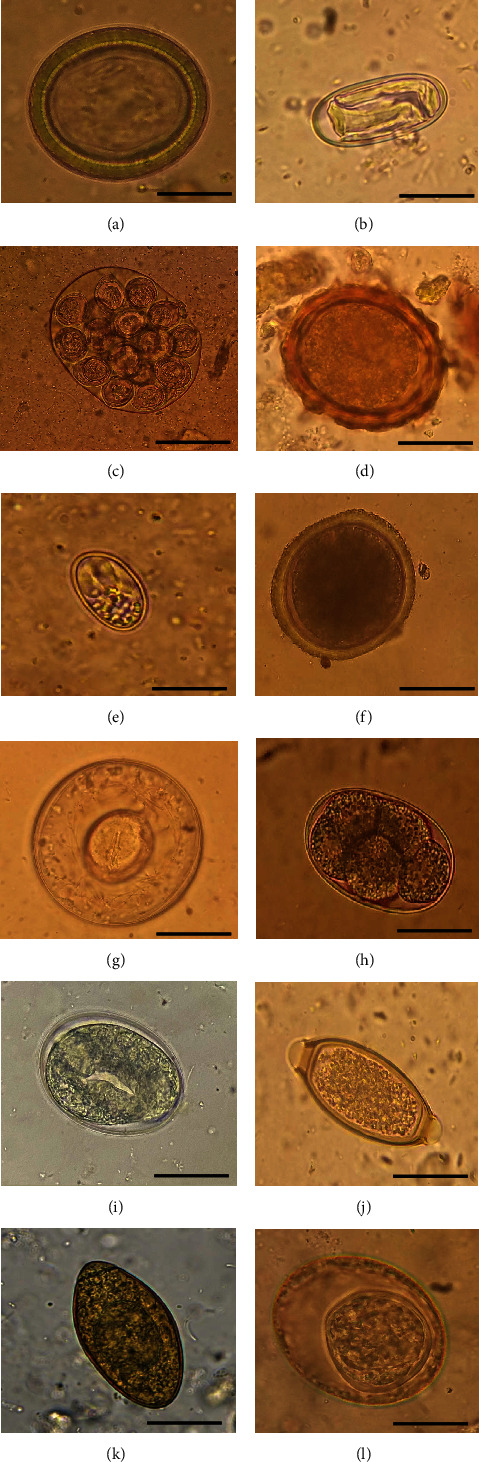
Common parasite eggs revealed from the soil and fecal samples collected in 12 villages in Northern Tanzania. (a) *Taeniid*, (b) *Spirocerca* sp. (c) *Dipylidium* eggs in an egg capsule, (d) *Ascaris* sp., (e) *Eimeria* sp., (f) *Toxocara* sp., (g) *Hymenolepis* sp., (h) hookworm, (i) *Strongyloides* sp., (j) *Trichuris* sp., (k) *Spirometra* sp., (l) *Toxascaris* sp. (scale: 100 *μ*m).

A total of 255 samples were collected from various origins, including latrines (*n* = 85), households (*n* = 58), and livestock enclosures housing sheep (*n* = 19), goats (*n* = 10), and cattle (*n* = 11), alongside samples from domestic dogs (*n* = 17) and chickens (*n* = 55). Distinct shapes of larvae and parasite eggs from cestodes, nematodes, and protozoans were observed based on the origin of the collected samples (Figure 1). Of the total sample collected, 152 tested positive with distinguished parasite eggs and oocysts. These parasites include hookworms (33/21.7%), *Trichuris* sp. (22/14.5%), *Strongyloides* sp. (21/13.8%), *Eimeria* sp. (30/19.7%), Taeniids (9/5.9%), *Hymenolepis* sp. (5/3.3%), *Spirometra* sp. (4/2.6%), and *Dipylidium* species (1/0.7%). Among the soil samples, 121 tested positive for hookworms (31/25.6%), *Trichuris* (22/18.2%), *Strongyloides* (19/15.7%), and *Eimeria* species (14/11.6%) (Table 3). On the other hand, 17 fecal samples from domestic dogs were observed with cestodes such as *Spirometra* species (4; (25.0%), Taeniids (2; 11.8%), *Hymenolepis* (2; 11.8%), hookworms (2; 11.8%), and *Spirocerca* species (Table 3). *Spirometra* species were predominantly identified from Arash Sokoni, Oloipiri, and Sukenya villages, while a significant number of Taeniids were found in Oloipiri, Wasso, Sukenya, Orkuyiene, Haytemba, and Loliondo villages (Table 4). Hookworms were most prevalent in Arash Sokoni (6/33; 18.2%), Loliondo (4/33; 12.2%), Sukenya, Isuguro, and Hyatemba (3/33; 9.1%) while *Strongyloides* were revealed in Wasso, Sukenya, Olobo, and Arash Sokoni villages with an average of 3/21 (14.3%) and *Eimeria* species in Orkuiyiene and Oloipiri villages with 6/30 (20%) and 5/30 (16.7%), respectively (Table 4). *Eimeria* species were found mainly from chicken in Orkuyiene (6/30; 20%), Oloipiri (5/30; 16.7%), Soitsambu, Wasso, and Gallapo villages (2/30; 6.7%) (Table 4).

The quantitative analysis of data reveals significant associations between various factors and the prevalence of STH across diverse villages in Tanzania. Key determinants such as hygiene practices, proximity to livestock enclosures, ecological conditions, and human–animal interaction emerge as pivotal influencers of zoonotic diseases and STH prevalence. For example, strong hygiene practices in Arash Sokoni and Soitsambu, indicated by a score of 7, correlate significantly with low *p* values of 0.0023 and 0.0067, respectively, highlighting a robust relationship between hygiene and STH prevalence (Table 5).

Similarly, communities residing closer to livestock enclosures (within 10–25 m) exhibit higher STH prevalence rates, as evidenced by significant chi-square values of 9.25, 10.85, 10.04, 7.69, and 5.58 in Arash Sokoni, Loliondo, Gallapo, Sukenya, and Haytemba villages, respectively. However, in Oloipiri, despite the close proximity to livestock enclosures, no significant value (*p* value = 3.20119*e*) is observed for parasitic infection and STH prevalence.

Ecological factors such as biodiversity and land use practices play a pivotal role in explaining STH prevalence, particularly evident in villages like Oloipiri, underscoring the significance of ecological conditions in shaping STH prevalence (Table 5). Additionally, human–animal interaction emerges as a significant factor, as observed in Isuguro and Gallapo, where scores of 8 and 9 correlate with significant chi-square values and low *p* values, highlighting the relevance of this factor in determining STH prevalence.

### 3.2. Understanding of Zoonosis

The understanding of zoonotic diseases and STHs among the surveyed population across different wards, namely, Oloirien Magaiduru, Oloipiri, Soitsambu, Arash, and Ololosokwan, was conducted. These wards are located in close proximity to protected areas, where human–wildlife interactions are common, including encounters with elephants (5.6%), buffalo (11.1%), deer (11.1%), wild pigs (5.6%), lions (11.1%), leopards (11.1%), reptiles (5.6%), rodents (16.6%), and wild birds (5.65%) (Table 6). The study also assessed the awareness of wildlife-related threats to human health, identifying coenurosis as a prevalent disease in the Loliondo area, while cysticercosis was more commonly reported in the Babati District. Moreover, diseases such as tuberculosis, anthrax, brucellosis, rabies, and taeniasis were mentioned as causing significant economic losses to the community. In terms of regional distribution, Arusha and Kilimanjaro emerged as the leading regions for reported zoonotic disease cases, with 22.9% and 17.1% prevalence, respectively, while Mwanza, Mbeya, Tanga, and Dar es Salaam regions reported only 11.4% combined, higher than the infection rate in Iringa Region (5.7%). Dodoma and Morogoro regions had the lowest reported rates, at 2.9% (Table 7). Additionally, 50% of respondents expressed the belief that government intervention is crucial in raising community awareness about zoonotic diseases, underscoring the importance of public health initiatives in mitigating these risks.

## 4. Discussion

This study focused on understanding and describing the prevalence of STHs within communities and its association with human–animal interactions. The study also explored health risk factors linked to activities conducted in proximity to protected areas. Results from this study highlight significant associations between various factors and the prevalence of STH across diverse villages. Notably, villages with higher hygiene practice scores, such as Arash Sokoni and Soitsambu, exhibited lower STH prevalence rates, aligning with prior research emphasizing the pivotal role of sanitation facilities and handwashing practices in mitigating STH transmission [28–32].

Additionally, communities residing closer to livestock enclosures (within 10–25 m) demonstrated higher STH prevalence rates, observed in Gallapo, Sukenya, Loliondo, and Haytemba villages, corroborating findings indicating that exposure to animal waste increases the risk of environmental contamination with STH eggs [33–35].

The results indicate that behavioral factors, such as open defecation or improper waste disposal, contribute to STH transmission, evidenced by elevated STH prevalence in villages with higher behavioral factor scores, including Oloipiri, Olobo, and Orkuyiene [36, 37]. We found that villages characterized by greater human–animal interaction, such as Isuguro and Gallapo, displayed heightened STH prevalence, highlighting the necessity of understanding zoonotic transmission routes in STH epidemiology [38, 39]. These findings emphasize the complex nature of STH transmission, influenced by environmental, behavioral, and ecological factors, necessitating comprehensive interventions to effectively address STH infections [40].

The geographical location and ecological context of each village played a pivotal role in shaping STH transmission dynamics. For instance, villages with higher ecological scores, such as Oloipiri and Soitsambu, demonstrated increased STH prevalence, likely attributable to favorable environmental conditions for parasite survival and transmission [31]. Additionally, the population density of wild and domesticated animals emerged as significant predictors, with higher densities associated with elevated STH prevalence rates, consistent with prior studies highlighting the role of animal reservoirs in sustaining STH transmission cycles [41, 42].

While this study offers valuable insights into zoonotic disease transmission dynamics, certain limitations deserve acknowledgment. The absence of infection intensity data restricts comprehensive characterization of parasitic infections. Moreover, the cross-sectional study design limits the ability to infer causal relationships between variables and comprehend temporal trends in parasitic prevalence. These limitations emphasize the need for further investigation to enhance the depth and rigor of future research endeavors in this field.

In conclusion, the findings from this study serve as baseline information to understand the zoonotic risks and the complex interplay between human behavior, environmental factors, and wildlife dynamics in driving STH transmission in Northern Tanzania.

We found that while fecal examination provides direct evidence of infection in hosts, soil examination allows for the identification of environmental contamination and potential sources of transmission. In our study, we observed significant associations between various human factors and STH prevalence, suggesting that human activities play a crucial role in shaping transmission dynamics. Addressing these multifactorial determinants requires integrated interventions that promote improved sanitation practices, enhance community awareness of zoonotic disease risks, and implement targeted control measures for both human and animal populations. By adopting a comprehensive One Health approach that considers the interconnectedness of human, animal, and environmental health, public health efforts can effectively mitigate the burden of STH infections and enhance overall community well-being.

## Figures and Tables

**Table 1:** Demographic and geographic characteristics of ethnic groups across different administration levels in the surveyed area.

**Study area**	**Village**	**Ward**	**District**	**Subvillage**	**Ethnic group**
Loliondo Division					
1	Wasso	Oloirien Magaiduru	Loliondo	Wasso	Maasai
2	Wasso	Oloirien Magaidutu	Loliondo	Olobo	Maasai
3	Soitsambu	Ololosokwan	Loliondo	Soitsambu	Maasai
4	Sukenya	Oloipiri	Loliondo	Embaash	Maasai
5	Orkuyiene	Oloipiri	Loliondo	Engutot	Maasai
6	Olobo	Oloipiri	Loliondo	Ormanie	Maasai
7	Isuguro	Soitsambu	Loliondo	Isuguro	Maasai
8	Loliondo	Orgosorok	Loliondo	Loliondo	Maasai
9	Arash Sokoni	Arash	Loliondo	Shuleni	Maasai
Babati District					
10	Ayasanda	Ayasanda	Babati	Ayasanda	Iraqw
11	Haytemba	Haytemba	Babati	Haytemba	Iraqw
12	Gallapo	Gallapo	Babati	Gallapo	Iraqw

**Table 2:** Demographic profile for participants in Focus Group Discussion.

**Variable**	**Categories (** **N** = **individual)**	**Loliondo**		**Babati**		**95% confidence interval**	**X** ^2^	**p** ** value**
		**Male** **n** **(%)**	**Female** **n** **(%)**	**Male** **n** **(%)**	**Female** **n** **(%)**			
Age	< 30 (*N* = 11)	5 (45.5)	2 (18.2)	3 (27.3)	1 (9.1)	0.3–11.7	0.88	0.35
	> 30 (*N* = 24)	11 (45.8)	6 (25)	4 (16.7)	3 (12.5)			

Occupation	Farmer (*N* = 12)	4 (33.3)	3 (25)	3 (25)	2 (16.7)	0.03–11.46	5.36	0.02
	Pastoralist (*N* = 23)	11 (47.8)	5 (21.7)	4 (17.4)	3 (13)			

Education	Primary level (*N* = 19)	9 (47.4)	4 (21.1)	3 (15.8)	3 (15.8)	0.58–2.42	3.33	0.07
	Secondary and tertiary (*N* = 6)	2 (33.3)	1 (16.7)	1 (16.7)	2 (33.3)			

**Table 3:** Parasite distribution in soil and fecal samples from livestock, dogs, and chickens collected in the selected villages in Northern Tanzania.

			**Soil samples**					**Fecal sample**	
			**Latrine (** **N** = 85**)**	**Household (** **N** = 58**)**	**Livestock enclosure (bomas)**			**Domestic dog (** **N** = 17**)**	**Chicken (** **N** = 55**)**
					**Sheep (** **N** = 19**)**	**Goat (** **N** = 10**)**	**Cattle (** **N** = 11**)**		
**Village surveyed**	**Egg/oocyst/larva**	**Size (*μ*m)**	**n** **(%)**	**n** **(%)**	**n** **(%)**	**n** **(%)**	**n** **(%)**	**n** **(%)**	**n** **(%)**
Village (*n* = 12)	*Ascaris* sp.	45–75 × 35–50	4 (5.8)	3 (12.0)	—	—	—	—	—
	*Strongyloides* sp.	50–58 × 20–35	9 (13.0)	6 (24.0)	2 (13.3)	2 (28.6)	3 (60)	2 (12.5)	
	*S. stercoralis* L1	280–310 × 14–16	5 (7.2)	—	—	—	—	—	—
	*Trichuris* sp.	50–58 × 22–27	13 (18.8)	3 (12.0)	6 (40)	3 (42.9)	—	—	—
	*Spirocerca* sp.	30–35 × 11–15	2 (2.9)	1 (4.0)	—	—	—	2 (12.5)	—
	*Toxocara* sp.	75 × 90	2 (2.9)	1 (4.0)	—	—	—	1 (6.3)	—
	*Enterobius* sp.	50–60 × 20–32	2 (2.9)	—	—	—	—	—	—
	Hookworms	60 × 40	15 (21.7)	7 (28.0)	5 (33.3)	2 (28.6)	—	2 (12.5)	—
	*Eimeria* sp.	21–42 × 16–9	8 (11.6)	4 (16.0)	2 (13.3)	—	—	1 (6.3)	15 (100.0)
	Taeniids	30–36	5 (7.2)	—	—	—	2 (40)	2 (12.5)	—
	*Hymenolepis* sp.		3 (4.3)	—	—	—	—	2 (12.5)	—
	*Dipylidium* sp.		—	—	—	—	—	1 (6.3)	—
	*Spirometra* sp.	58 × 45	—	—	—	—	—	4 (25.0)	—

Abbreviations: % = percentage for positive samples, *N* = total number of sample collection, *n* = number of positive samples.

**Table 4:** Parasite egg distribution and prevalence of parasitic organisms within the study area.

**Village**	**No. of sample (** **N** **)**	**Positive (** **n** **)**	**Prevalence (** **n**/**N**∗100**)**	**Parasite egg or oocysts observed**												
				***Ascaris* sp.**	***Strongyloides* sp.**	***S. stercoralis***	***Trichuris* sp.**	**Hookworms**	***Eimeria* sp.**	**Taeniids**	***Hymenolepis* sp.**	***Dipylidium* sp.**	***Spirometra* sp.**	***Spirocerca* sp.**	***Toxocara* sp.**	***Enterobius* sp.**
Oloipiri	28	20	0.71	—	1	1	4	2	5	2	—	—	1	1	1	2
Wasso	16	12	0.75	1	3	—	2	2	3	1	—	—	—	—	—	—
Soitsambu	21	12	0.57	1	1	1	1	2	3	—	—	—	—	2	1	—
Sukenya	22	14	0.64	—	3	2	—	3	2	1	1	—	1	1	—	—
Orkuyiene	21	16	0.76	2	2	—	2	2	6	1	—	—	—	1	—	—
Olobo	18	12	0.67	1	3	—	2	2	3	—	1	—	—	—	—	—
Isuguro	17	9	0.53	—	1	—	2	3	1	1	1	—	—	—	—	—
Loliondo	23	14	0.61	1	2	1	2	4	1	1	1	—	—	1	—	—
Arash Sokoni	43	17	0.40	—	3	—	2	6	1	1	—	1	2	—	1	
Ayasanda	18	9	0.50	1	1	—	2	2	2	—	—	—	—	1	—	—
Haytemba	15	9	0.60	—	—	—	2	3	1	1	1	—	—	—	1	—
Gallapo	13	8	0.62	2	1	—	1	2	2	—	—	—	—	—	—	—
Total	255	152		9	21	5	22	33	30	9	5	1	4	7	4	2

**Table 5:** Association of community lifestyle and risks on zoonosis and STH in the selected villages adjacent to the protected areas.

**Village**	**Hygiene practices (score)**	**Proximity to livestock enclosure (meters)**	**Behavioral factor (score)**	**Ecological factor (score)**	**Human–animal interaction (score)**	**Population density of wild animals**	**Population density of domesticated animals**	**Species diversity (score)**	**Knowledge about STH or zoonotic potential (score)**	**X** ^2^	**DF**	**95% confidence interval**	**p** ** value**
Oloipiri	8	25	7	9	7	18	50	9	8	107.65	1	7.34–20.43	3.20119*e*‐25
Wasso	4	30	5	7	4	15	40	7	4	8.87	1	0.02–1.95	0.002896279
Soitsambu	7	15	6	9	5	30	55	8	7	7.33	1	0.37–2.00	0.00677508
Sukenya	5	25	4	6	7	10	50	5	6	7.69	1	0.43–2.14	0.00555613
Orkuyiene	6	20	8	6	7	30	60	6	5	4.90	1	0.09–0.10	0.026901521
Olobo	9	50	7	8	6	35	45	7	4	19.28	1	0.61–4.89	1.12677*e*‐05
Isuguro	5	30	4	5	8	40	65	5	8	9.36	1	0.07–2.01	0.00221191
Loliondo	6	20	6	3	4	6	40	4	6	10.85	1	0.35–2.76	0.000987648
Arash Sokoni	7	10	6	8	5	22	48	8	5	9.25	1	0.57–2.63	0.002356767
Ayasanda	6	40	5	7	6	28	52	6	6	6.14	1	0.38–1.74	0.013252868
Haytemba	8	25	7	9	7	18	42	9	8	5.58	1	0.33–0.90	0.018127454
Gallapo	5	15	4	6	9	32	58	7	6	10.04	1	0.08–2.31	0.001535689

*Note:* Score range: low range: 1–5; high range: 6–10.

Abbreviation: *X*^2^ ^=^ chi-square test.

**Table 6:** Key informants' insights on wildlife interaction with domestic animals and potential zoonotic disease risks.

**Animal species involved**	**Domestic animal involved**	**Possible zoonotic diseases**	**Rate (respondents)**
Elephant, buffalo	Cattle, goat, sheep	Tuberculosis, anthrax	High
Buffalo, deer	Cattle, goat, sheep	Tuberculosis, brucellosis	High
Wild dog, bats	Dogs	Rabies	High
Zebra, antelopes	Sheep, cattle, goat	Tuberculosis	Moderate
Reptiles (snakes)	Livestock, chicken	Avian influenza, salmonellosis	Low
Rodents, rabbits, deer, antelopes	Cattle, sheep, goats	Leptospirosis	High
Rodents	Dogs, cattle, pigs	Leptospirosis	Moderate
Wild birds	Chicken	Avian influenza, salmonellosis	Low
Rodents, birds	Cats, livestock		Low
Wild pigs, antelopes	Pigs	Cysticercosis	Moderate
Lion, leopard, squirrels	Dog	Coenurosis	Low
Lion, leopard	Cattle, pigs	Coenurosis, cysticercosis	High

**Table 7:** Key informant response on zoonotic disease types, animal species involved, and geographic locations in Tanzania.

**Type of zoonotic disease**	**Ethnic name**	**Causative agent**	**Animal species involved**		**Possible location of outbreak in Tanzania**
			**Wildlife species**	**Domestic animal**	
Anthrax	Nkuguaenkaji (Maasai)	*Bacillus anthracis*	Elephant, buffalo	Cattle, goat, sheep	Arusha, Kilimanjaro, and Tanga
Brucellosis	Olmekenyu (Maasai)	*Brucella* sp.	Buffalo, deer	Cattle, goat, sheep	Arusha, Dodoma, and Iringa
Rabies	Olondusero (Maasai)	Lyssavirus	Wild dog, bats	Dogs	Dar es Salaam, Mwanza, and Mbeya
Rift Valley fever	Hom ya bonde la ufa (Swahili)	Phlebovirus	Buffalo, zebra, antelopes	Sheep, cattle, goat, camels	Morogoro, Pwani, and Tanga
Salmonellosis	Salmonella (Swahili)	*Salmonella* sp.	Reptiles (snakes)	Livestock, poultry (chicken, pigeon)	Dar es Salaam, Mwanza, and Tanga
Avian influenza	Homa ya kuku (Swahili)	Influenza A virus	Wild birds	Wild birds, domesticated poultry	Kilimanjaro, Mbeya, and Tanga
Toxoplasmosis		*Toxoplasma gondii*	Rodents, birds	Cats, livestock	Dar es Salaam, Arusha, and Mwanza
Cysticercosis	Homa ya nguruwe (Swahili)	*Taenia solium*	Wild pigs, antelopes	Pigs	Iringa, Mbeya, and Songwe regions
Echinococcosis	Homa ya mapafu (Swahili)	Tapeworm	Lion, leopard, squirrels	Pigs	Arusha, Iringa
Taeniasis	Minyoo (Swahili)	*Taenia saginata*, *Taenia solium*	Wild animals	Cattle, pigs	Arusha, Kilimanjaro, and Manyara regions
Cysticercosis, taeniasis		*Taenia solium*	Wild animals	Livestock, wild mammals, pig	Arusha, Kilimanjaro, and Manyara regions
Coenurosis	Omillo (Maasai)	*Taenia multiceps*	Wild animals	Dogs, sheep, goat	Arusha, Kilimanjaro, and Manyara regions

## Data Availability

All data are incorporated in the manuscript.
